# Supplementary material for: Scale-invariant large nonlocality in polycrystalline graphene
Source: Nat Commun. 2017 Dec 19;8:2198. doi: 10.1038/s41467-017-02346-x (PMC5736749; doi:10.1038/s41467-017-02346-x)
Supplement: Supplementary file 1 — Supplementary Information [file 41467_2017_2346_MOESM1_ESM.pdf]

Supplementary Note 1: Preliminary electrical characterization of chemical vapour deposition graphene devices.

Standard electrical characterization was performed in the different chemical vapour deposition (CVD) graphene Hall bars. Supplementary Figure 1 shows *I-V* curves, transfer curves, temperature dependence of the sheet resistance (ρ_{xx}), and the temperature dependence of the carrier density ($n_{2\mathrm{D}}$) obtained from Hall measurements for the 500-µm-wide sample. Supplementary Table 1 shows the mobility determined from the expression $\mu = \frac{1}{\rho_{xx} n_{2\mathrm{D}} e}$, where ρ_{xx} is taken for a doping of 1×10^{12} cm^{-2}, and ρ_{xx} at the charge neutrality point (CNP) for the samples shown in the main manuscript.

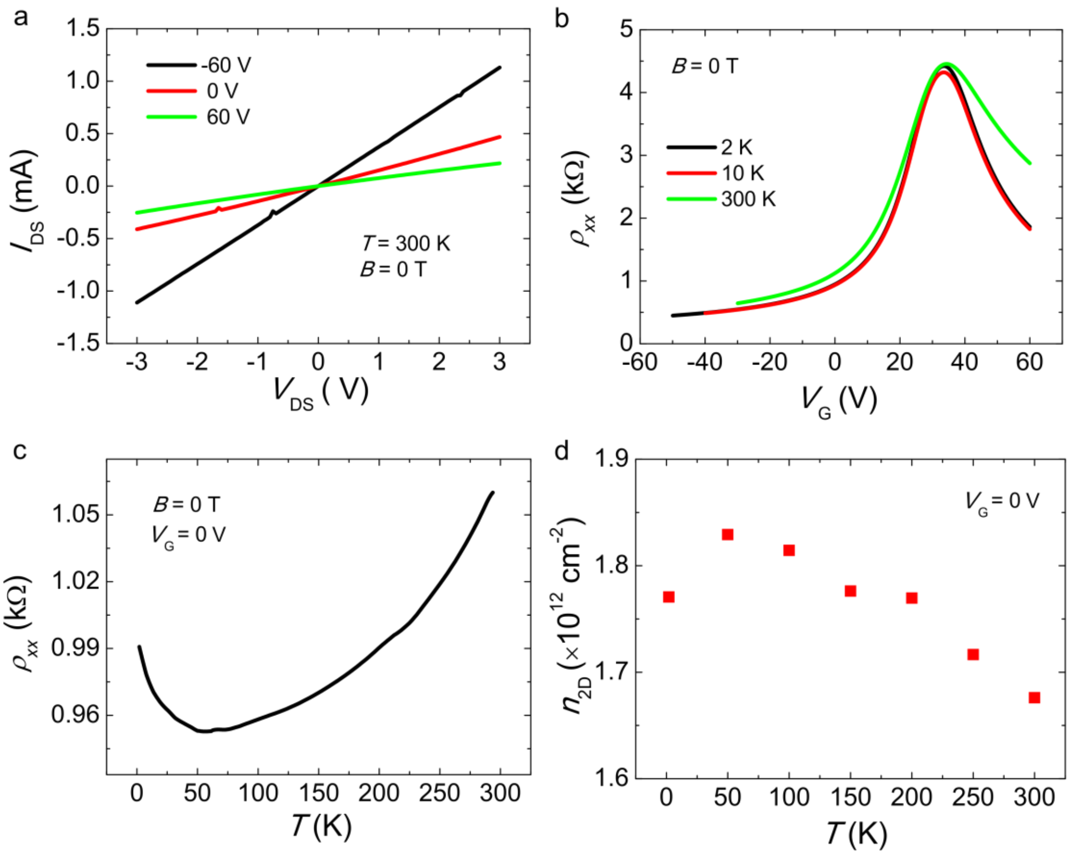

Supplementary Figure 1 | Typical electrical performance of the devices fabricated. a) Two-point output characteristics of the 500-µm-wide CVD graphene sample contacted with Ti (5 nm)/Au (40 nm) showing linear *I-V* curves. b) Sheet resistance as a function of gate voltage (transfer characteristics) of the 500-µm-wide CVD graphene device with *L/W* = 3 after the in-situ annealing at 400 K with helium flushes, at several temperatures. Thickness of the SiO_2 dielectric is 300 nm. c) Temperature dependence of the sheet resistance of the 500-µm-wide CVD graphene device with *L/W* = 3 at V_G = 0 V, with weak-localization effects emerging for the lowest temperatures. d) Temperature dependence of the carrier density determined from Hall measurements of the 500-µm-wide CVD graphene device with *L/W* = 3 at V_G = 0 V.

Supplementary Table 1 | Sheet resistance at the CNP and mobility for the devices shown in the manuscript at a temperature of 2 K.

**Channel width (µm)**	**500**	**50**	**5**
**μ ($\mathrm{cm}^2\ \mathrm{V}^{-1}\ \mathrm{s}^{-1}$) @ 2 K**	2653	3120	3870
**ρ_{xx} @ 2 K @ CNP (Ω)**	4410	4319	3957

Supplementary Note 2: Magnetotransport measurements of chemical vapour deposition graphene devices.

We performed magnetotransport measurements of the CVD graphene Hall bars in order to characterize their edge transport. Supplementary Figure 2 shows the half-integer quantum Hall effect (QHE) well developed for an external magnetic field of 9 T for the whole range of device dimensions explored in our study.

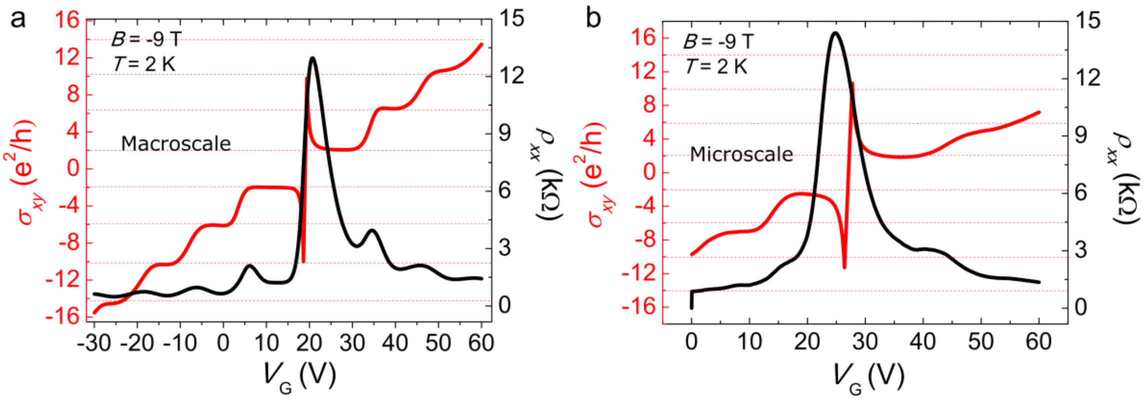

Supplementary Figure 2 | Half-integer quantum Hall Effect in the CVD graphene samples. a) Transverse conductivity σ_{xy} and sheet resistance ρ_{xx} as a function of the gate voltage for the 500-μm-wide macroscale samples. b) σ_{xy} and ρ_{xx} as a function of the gate voltage for the 5-μm-wide microscale samples. Dashed lines represent the quantized transverse conductivity according to $\sigma_{xy} = \pm 4(i + 1/2)\frac{e^2}{h}$, with *i* = 0, 1, 2, …

Supplementary Note 3: Temperature dependence of the nonlocality for CVD graphene devices.

While the study of the nonlocal resistance was mainly performed at the lowest temperatures available to our system (*T* = 2 K), the effects are visible for the entire range of temperatures up to room temperature. Supplementary Figure 3 shows the nonlocal resistance as a function of the gate voltage for different temperatures between 2 K and 300 K, for both macroscale and microscale devices.

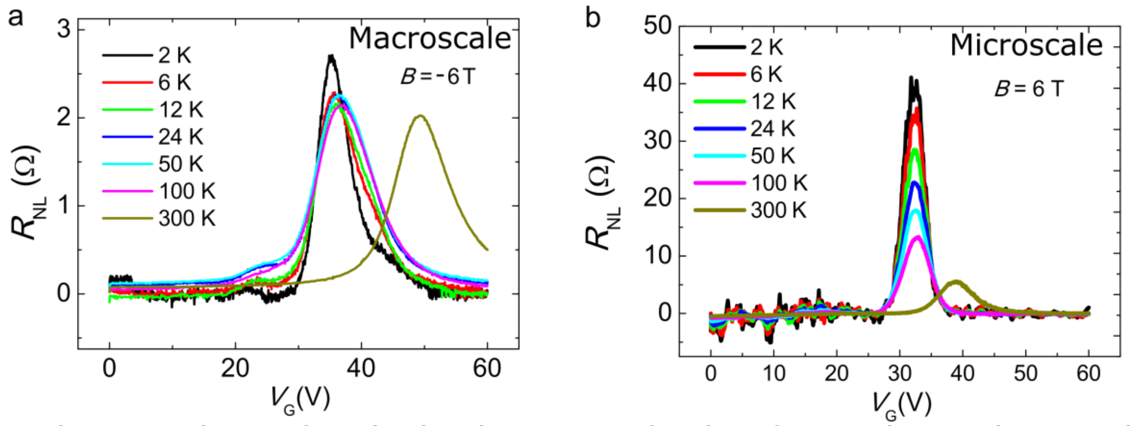

Supplementary Figure 3 | Nonlocal resistance as a function of gate voltage under external magnetic field magnitude of 6 T for several temperatures. a) 500-μm-wide macroscale device with *L*/*W* = 3. b) 5-μm-wide microscale device with *L*/*W* = 3.2. In both cases, the shift in the CNP for the 300 K measurements is related to the adsorption of water (see Methods in the main manuscript).

Supplementary Note 4: Magnetic field asymmetry in nonlocal resistances

In this note, we provide a simple Landauer-Büttiker analysis of how nonlocal resistances can develop an asymmetry with respect to the sign of an applied magnetic field. We consider a four-probe device with three different device, grain boundary (GB) and backscattering configurations.

Quantum spin Hall limit. Firstly, we consider the spin-split QHE induced by Zeeman splitting at the CNP in the absence of GBs or scattering. In this case, perfectly ballistic counter-propagating edge states allow unitary transmission between neighbouring probes and give rise to an effective quantum spin Hall effect (QSHE). This case, shown schematically in Supplementary Fig. 4, gives rise to a quantized nonlocal resistance. A nonlocal resistance arises solely due to potential drops at the probes, and the quantized R_{NL} value is dependent on the device geometry and probe layout.

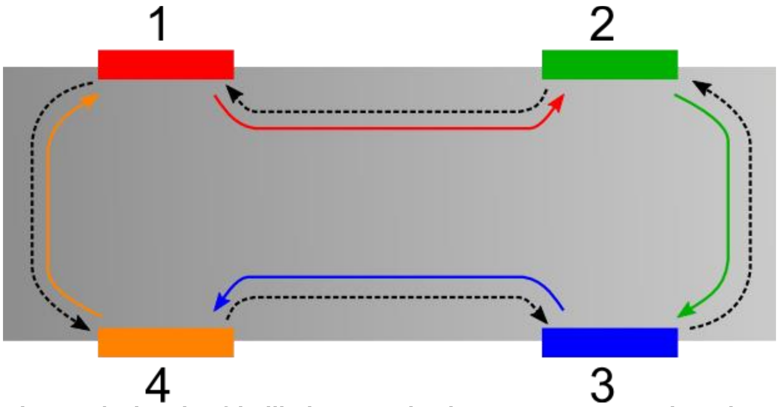

Supplementary Figure 4| Sketch of ballistic, quantized, counter-propagating channels in Zeeman-split QHE. Solid arrows show electron channels, colored by source probe, and dashed black arrows shows the hole channels. Arrow directions are inverted upon changing the sign of the external magnetic field *B*.

At the CNP, up and down spins are carried by separate electron and hole channels. The transmission matrices for electrons and holes here are given by $T^{\mathrm{e}} = \begin{pmatrix} 0 & 0 & 0 & 1 \\ 1 & 0 & 0 & 0 \\ 0 & 1 & 0 & 0 \\ 0 & 0 & 1 & 0 \end{pmatrix}$ and $T^{\mathrm{h}} = \begin{pmatrix} 0 & 1 & 0 & 0 \\ 0 & 0 & 1 & 0 \\ 0 & 0 & 0 & 1 \\ 1 & 0 & 0 & 0 \end{pmatrix}$ respectively, where T^{e}_{pq} describes transmission from probe q to probe p due to electrons. As the spin channels are independent, the total transmission is a simple sum of these terms, giving $T = \begin{pmatrix} 0 & 1 & 0 & 1 \\ 1 & 0 & 1 & 0 \\ 0 & 1 & 0 & 1 \\ 1 & 0 & 1 & 0 \end{pmatrix}$.

The nonlocal resistance is determined by solving the multi-terminal Landauer formula $I_p = \frac{e^2}{h}\sum_q \left(T_{qp}V_p - T_{pq}V_q\right)$ for the currents and potentials at each probe, and in this case is found to be $R_{\mathrm{NL}} = \frac{1}{4}\frac{h}{e^2}$. Changing the sign of B changes the direction of current flow for both the electron and hole channels, and is equivalent to taking the transpose of T. Since T is symmetric, the nonlocal resistance is unchanged.

Grain boundary with electron-hole asymmetric transmission. We now consider the simple case where the GB has a transmission channel for electrons only. This is easily generalizable to

the case where the GB transmits electrons and holes with different probabilities. We also assume that one end of the GB is located near one of the contacts (probe 3) so that direct transmission from the GB to the contact is possible – we will later explore the relaxation of this assumption. The setup, as shown in Supplementary Fig. 5, differs from the previous case only for the spin channel carried by electrons: e.g. a certain amount (‘x’) of electron current from probe 1 towards probe 2 is now deflected along the grain boundary, where it again splits with part (‘y’) travelling along the electron edge channel to probe 3, and the remainder (‘x-y’) coupling directly to probe 4. Electron current from probe 3 experiences a similar split. The transmission matrix here is $T = \begin{pmatrix} 0 & 1 & 0 & 1 \\ 1-x & 0 & x+1 & 0 \\ x-y & 1 & 0 & 1 \\ y+1 & 0 & 1-y & 0 \end{pmatrix}$, giving nonlocal resistances $R_{\mathrm{NL}}(B > 0) = \frac{1-x}{4+2x-2y}\frac{h}{e^2}$ and $R_{\mathrm{NL}}(B < 0) = \frac{1-y}{4+2x-2y}\frac{h}{e^2}$.

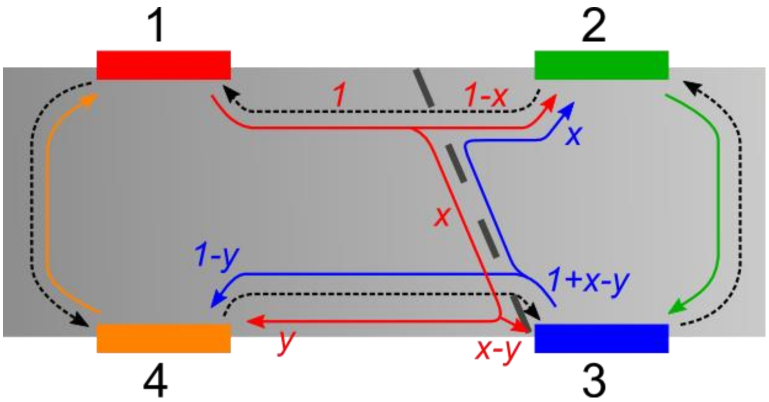

Supplementary Figure 5 | Sketch for Zeeman-split QHE system with a grain boundary allowing transmission only for electrons. As in Supplementary Fig. 4, but with a grain boundary connecting the two sides of the device and allowing electron transmission. The red and blue labels show the division of current in the electron channels emerging from leads 1 and 3 due to the grain boundary.

The difference between these values depends strongly on a direct coupling between the GB and one of the probes – as this is switched off $(y \to x)$, the asymmetry vanishes despite changes in the individual potentials, and despite T not being a symmetric matrix. An asymmetric T is a necessary but not sufficient condition for a B-field asymmetry – in the QHE, T is asymmetric but the nonlocal resistance is zero for both charge carrier types. However, we also note that electron-hole asymmetry is essential to the appearance of a B-field asymmetry – equal transmission for elections and holes through the GB results in a symmetric T matrix where changing the sign of B merely swaps the roles of electrons and holes while conserving the total transmissions.

So, even in the limit of a quasi-QSHE with perfectly ballistic edge channels, an asymmetric non-local response can emerge due to the presence of a GB supporting electron-hole asymmetric transmission and a direct coupling to one of the probes. In order to directly couple to a probe the separation between the GB and the probe should be on the order of the magnetic length l_B, which for the experimental case at $B = 6$ T is ~10 nm. This length may be extended due to, e.g., electron-hole puddles which can open additional conducting channels near *pn* interfaces, but is most likely too small to account for all the experimental signatures, in particular for the largest systems. However, it is a good system to test electron-hole nonlocal asymmetries within a tight-binding framework, and we shall examine it further in Supplementary Note 5.

Electron-hole asymmetric grain boundaries with backscattering. We now move from the effective QSHE regime to consider the dissipative regime where the edge channels are no longer ballistic. As discussed in Abanin *et al.* (see Supplementary Ref. 1), this can occur by

backscattering along one edge or through bulk-mediated mechanisms. Backscattering between channels along the same edge requires mixing of the different spin channels, which can be mediated by spin-orbit coupling or magnetic disorder. In this situation, we consider a very simple example where backscattering reduces transmission along the top edge of the device by an amount a for both electrons and holes (see Supplementary Fig. 6). This can be easily generalised to cases with different backscattering rates for different edges and carrier types to account for, e.g., the distance dependence of transmissions through systems with scattering. The grain boundary once more transmits only for electrons, but this time does not have a direct coupling to any lead. We now have $T = \begin{pmatrix} 0 & 1-a & 0 & 1 \\ 1-a-x & 0 & x+1 & 0 \\ 0 & 1 & 0 & 1 \\ 1+x & 0 & 1-x & 0 \end{pmatrix}$, $R_{\mathrm{NL}}(B>0) = \frac{1-a-x}{4-a(x+3)}\frac{h}{e^2}$ and $R_{\mathrm{NL}}(B<0) = \frac{(1-a)(1-x)}{4-a(x+3)}\frac{h}{e^2}$. As before, the asymmetry between B and -B only arises when an electron-hole asymmetric channel opens between the two sides of the device.

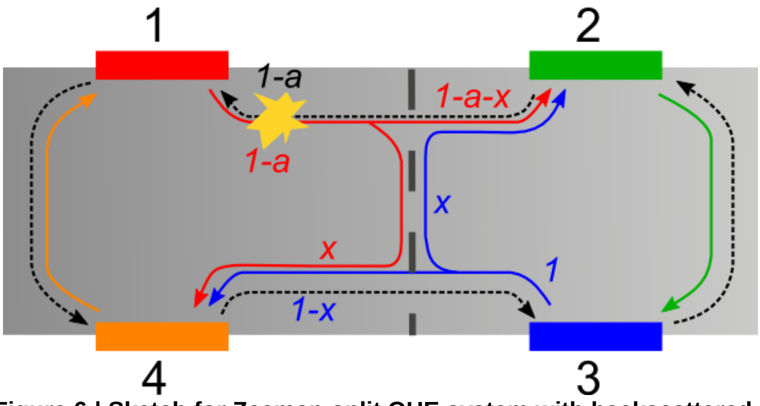

Supplementary Figure 6 | Sketch for Zeeman-split QHE system with backscattered channels and an electron-hole asymmetric grain boundary. As in Supplementary Fig. 5, but now the grain boundary has no direct connection to any probe. Electrons and hole channels between probes 1 and 2 are now dissipative, with backscattering (e.g. the yellow region) reducing the total current in these channels to *1-a*.

Supplementary Note 5: Tight-binding calculations

The setup for the tight-binding simulation is shown in Supplementary Fig. 7a. We consider a ~ 40-nm-wide 85-nm-long armchair nanoribbon with four metallic probes attached as shown. A 558-grain boundary (GB) is placed between the current and voltage probes, and we displace probe 3 so that it can couple directly with the GB as discussed in the previous section. To model a disordered GB as expected in experiment, Anderson disorder is added in a narrow channel surrounding the GB (shown by red and blue circles in Supplementary Fig. 7a for positive and negative potentials, respectively). In addition, Gaussian-type electron-hole puddles are included throughout the device to simulate the effects of charged defects in the substrate. An example of the net potential profile in the system is shown by the color map in Supplementary Fig. 7b.

The interprobe transmissions are calculated using the Landauer formula with recursive Green's functions techniques. From these the nonlocal resistance is calculated. The two effects of an external, perpendicular magnetic field are considered separately within our simulations.

Firstly, the onset of a stable quantum Hall regime is controlled by including Peierl's phase factors in the tight-binding hopping parameters. Since our simulated systems have significantly narrower width than the experimental cases, we chose B = ±60 T which ensures that the edge states on either side of the device are sufficiently decoupled. A Zeeman splitting is then manually introduced to break the degeneracy between up- and down-spin states. We choose $\Delta Z \sim 10^{-3}$ eV, agreeing with the expected values, including interaction effects, for an experimental $B \sim 10$ T (see Supplementary Ref. 1). This is applied as a rigid shift of the two spin bands of

±ΔZ/2. Similar effects to those discussed in the main paper, and below, can be achieved with a variety of different B and ΔZ values.

To test our simulation approach, we calculate the nonlocal resistance for a pristine ribbon without a GB or potential disorder. A nonlocal resistance peak at the CNP is observed with a height slightly below the value $R_{\mathrm{NL}} = \frac{1}{4}\frac{h}{e^2}$ expected in the QSHE regime. Supplementary Figure 7c shows this system with an exaggerated ΔZ to highlight that the peak width agrees exactly with the Zeeman splitting. The minor deviation from the quantized value is due to quantum tunneling processes through the bulk of the narrow ribbon simulated – this deviation decreases on increasing the magnetic field or the Zeeman splitting as expected. Furthermore, the curves for positive and negative B coincide for this system. Additional peak features at $E_{\mathrm{F}} = \pm 0.09|t|$ are due to the bulk states of the 1st Landau level (LL1), which are broadened and split due to the Zeeman term. Features occur at this position for all our simulated systems, and at the expected LL1 positions in the experimental results for the microscale devices. We note that these peaks emerge due to a nonlocal signal driven by bulk states, unlike the edge state processes determining the signal at the CNP.

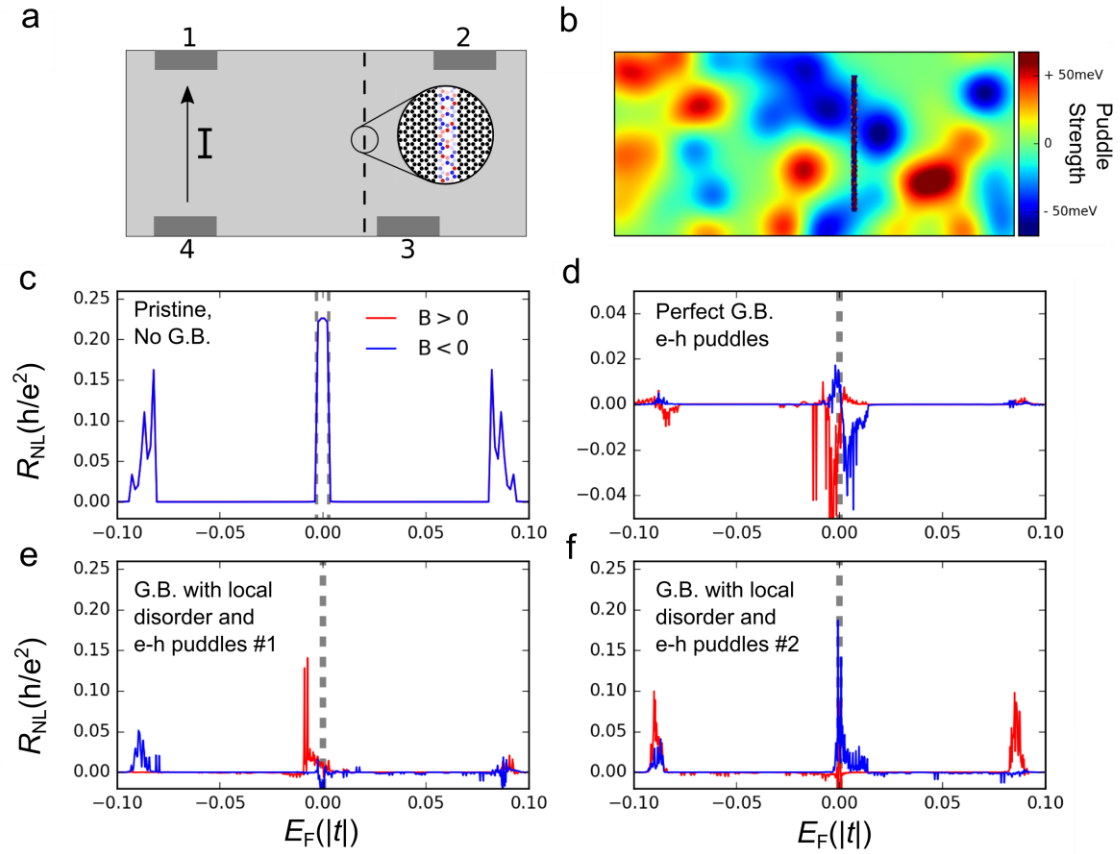

Supplementary Figure 7 | Tight-binding calculations. a) Sketch of the 4-probe device setup for the tight-binding simulation. A current is driven between probes 4 and 1, and the nonlocal resistance is measured between probes 3 and 2. A disordered 558-type grain boundary connects the two sides of the device. b) An example of the potential profile due to both electron-hole puddles and local Anderson disorder on the grain boundary sites. c-f) Examples of R_{NL} calculated for different systems, with the width of the Zeeman splitting in each case shown by dashed lines. c) shows a pristine ribbon device, d) introduces a perfect 558-GB without Anderson disorder, but with electron-hole puddles. e) and f) show two examples with Anderson-disordered GBs and electron-hole puddles. In all cases, B is perpendicular to the sample plane and has magnitude of 60 T.

Supplementary Figure 7d shows the dramatic effect that a highly conducting GB has on the nonlocal signal. The pristine GB introduces a direct channel between the current probes,

shunting the edge state channels which underpin the R_{NL} peak at the CNP. The resulting signal is highly asymmetric for different signs of B and oscillates around zero. The range of non-zero values is broadened by the introduction of electron-hole puddles.

Realistic CVD devices are unlikely to contain long regions of atomically pristine GBs, so we add a strong Anderson-type disorder of strength $3t$ to atomic sites within 2 lattice constants of the grain boundary. This reduces the transmission efficiency of the bulk leakage channel, and allows us to tune the relative contributions of the counter-propagating edge states and grain boundary channels. Two examples with different background puddle configurations are shown in panels in Supplementary Fig. 7e and Supplementary Fig. 7f. These simulations replicate the main features of the experimental data – a large peak near the CNP for one sign of B and an almost complete suppression of the peak for the opposite sign. Furthermore, we note that the experimental features observed at LL1 in the microscale devices are also observed here. Comparing the two examples, we note that the sign of B displaying the peak depends on the underlying electron-hole puddle distribution. The distribution corresponding to Supplementary Fig. 7e is shown in Supplementary Fig. 7b, where we can expect that the transmission of the GB will be strongly affected by the two hole-doped regions through which is passes. Indeed, very large puddles capable of spanning the device can set up leakage channels through the formation of snake states along *pn* boundaries. However, this is unlikely in the experimental setup due to a device width much larger than the puddle length scale (~10 nm).

Supplementary Note 6: Dependence of the nonlocality with the sign of the magnetic field for different aspect ratios and sample widths.

The strong asymmetry of the nonlocal signal with the sign of the magnetic field was present for all the devices considered in the study. For the 5-μm-wide microscale sample, the suppression of the nonlocal signal at the CNP is more evident. Supplementary Figure 8 shows the nonlocal resistance as a function of the gate voltage for all the devices in the 5-μm-wide microscale sample, and for one device in the 500-μm-wide macroscale sample.

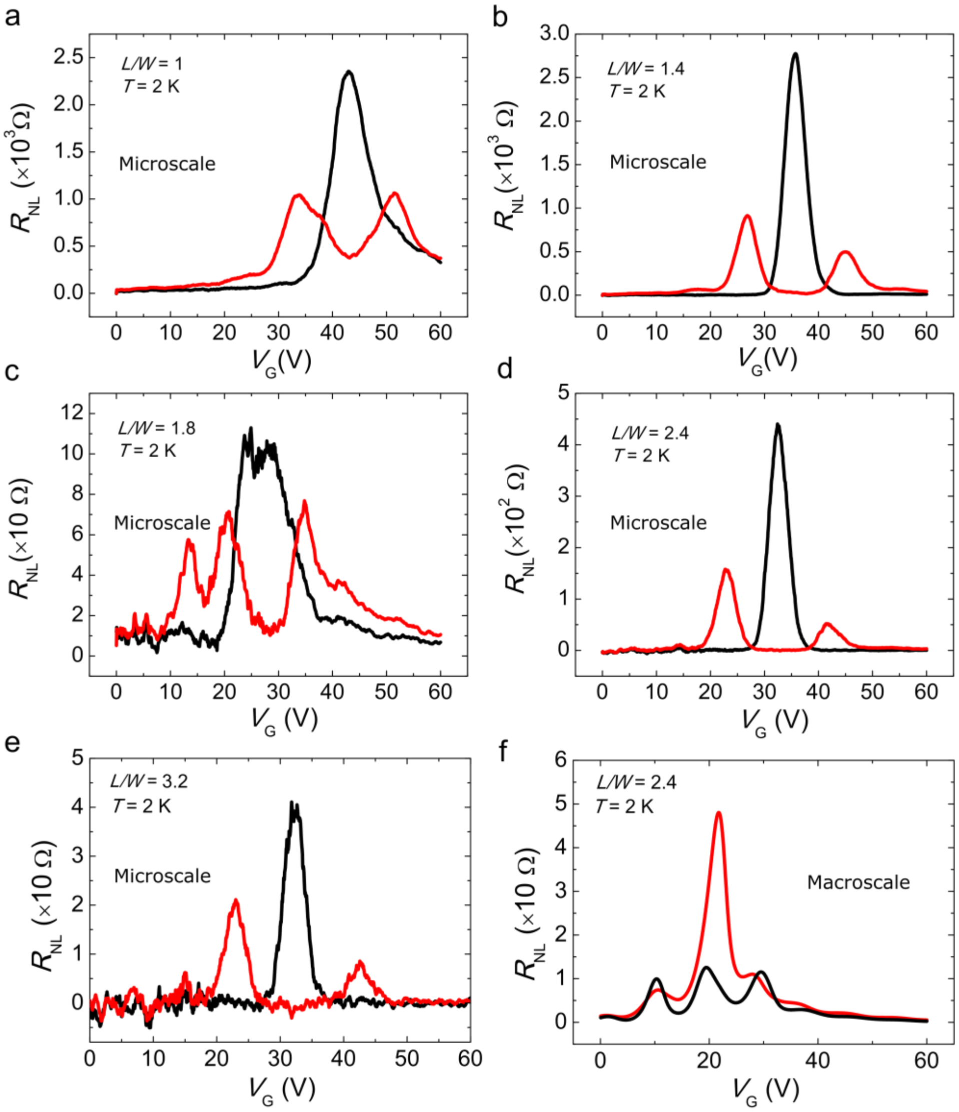

Supplementary Figure 8 | Nonlocal resistance as a function of the gate voltage for opposite signs of an external magnetic field with magnitude of 6 T for several aspect ratios and channel widths. Black solid lines: B = +6 T, Red solid lines: B = - 6 T. Panels a), b), c), d), and e) correspond to the 5-μm-wide sample, with L/W = 1, 1.4, 1.8, 2.4, and 3.2, respectively. Panel f) corresponds to the 500-μm-wide sample, with L/W = 2.4.

Supplementary References

1. Abanin, D. A., Lee, P. A. & Levitov, L. S. Spin-Filtered Edge States and Quantum Hall Effect in Graphene. *Phys. Rev. Lett.* **96,** 176803 (2006).
